# The Impact of Cardiac Comorbidity Sequence at Baseline and Mortality Risk in Type 2 Diabetes Mellitus: A Retrospective Population-Based Cohort Study

**DOI:** 10.3390/life12121956

**Published:** 2022-11-22

**Authors:** Sharen Lee, Helen Huang, Teddy Tai Loy Lee, Cheuk To Chung, Oscar Hou In Chou, Keith Sai Kit Leung, Abraham Ka Chung Wai, Wing Tak Wong, Tong Liu, Carlin Chang, Gary Tse

**Affiliations:** 1 Diabetes Research Unit, Cardiovascular Analytics Group, Hong Kong, China; 2 Department of Medicine, Queen Mary Hospital, University of Hong Kong, Hong Kong, China; 3 Emergency Medicine Unit, Li Ka Shing Faculty of Medicine, The University of Hong Kong, Hong Kong, China; 4 School of Life Sciences, Chinese University of Hong Kong, Hong Kong, China; 5 Tianjin Key Laboratory of Ionic-Molecular Function of Cardiovascular Disease, Department of Cardiology, Tianjin Institute of Cardiology, Second Hospital of Tianjin Medical University, Tianjin 300211, China; 6 Kent and Medway Medical School, Canterbury CT2 7NT, UK

**Keywords:** comorbidity, sequence, all-cause mortality, medication

## Abstract

Introduction: The presence of multiple comorbidities increases the risk of all-cause mortality, but the effects of the comorbidity sequence before the baseline date on mortality remain unexplored. This study investigated the relationship between coronary heart disease (CHD), atrial fibrillation (AF) and heart failure (HF) through their sequence of development and the effect on all-cause mortality risk in type 2 diabetes mellitus. Methods: This study included patients with type 2 diabetes mellitus prescribed antidiabetic/cardiovascular medications in public hospitals of Hong Kong between 1 January 2009 and 31 December 2009, with follow-up until death or 31 December 2019. The Cox regression was used to identify comorbidity sequences predicting all-cause mortality in patients with different medication subgroups. Results: A total of 249,291 patients (age: 66.0 ± 12.4 years, 47.4% male) were included. At baseline, 7564, 10,900 and 25,589 patients had AF, HF and CHD, respectively. Over follow-up (3524 ± 1218 days), 85,870 patients died (mortality rate: 35.7 per 1000 person-years). Sulphonylurea users with CHD developing later and insulin users with CHD developing earlier in the disease course had lower mortality risks. Amongst insulin users with two of the three comorbidities, those with CHD with preceding AF (hazard ratio (HR): 3.06, 95% CI: [2.60–3.61], *p* < 0.001) or HF (HR: 3.84 [3.47–4.24], *p* < 0.001) had a higher mortality. In users of lipid-lowering agents with all three comorbidities, those with preceding AF had a higher risk of mortality (AF-CHD-HF: HR: 3.22, [2.24–4.61], *p* < 0.001; AF-HF-CHD: HR: 3.71, [2.66–5.16], *p* < 0.001). Conclusions: The sequence of comorbidity development affects the risk of all-cause mortality to varying degrees in diabetic patients on different antidiabetic/cardiovascular medications.

## 1. Introduction

The presence of multiple comorbidities is a known risk factor for all-cause mortality across the spectrum of illnesses [1,2,3,4,5,6]. However, the effects of the sequence of comorbidity development on patient mortality remain unexplored. The difference in comorbidity sequences can reflect varying disease severity, disease phenotypes and, possibly, a different disease prognosis, which may require alterations to the management approach. Additionally, the temporal variability of risk factors has been increasingly recognized as a prognostic marker for the progression of chronic diseases [7,8]. For example, long-term glycemic and lipid variability have been used to predict cardiovascular, renal and mortality in type 2 diabetes mellitus [9,10]. The sequence of comorbidity development can be viewed as another form of long-term temporal variability in the course of the chronic disease, and therefore, has a prognostic potential.

In type 2 diabetes mellitus, a metabolic syndrome that is becoming increasingly prevalent due to prolonged life expectancy under a sedentary lifestyle, macrovascular and microvascular complications often arise along its disease course. Coronary heart disease (CHD), atrial fibrillation (AF) and heart failure (HF) are three common cardiovascular complications that have an interacting and interdependent relationship in their pathogenesis and progression. Because diabetic patients are at a higher risk of adverse cardiovascular events, the employment of antidiabetic agents and regimens often plays a crucial role in cardiovascular prevention by treating clinical features of metabolic syndrome [11]. The combination of cardiovascular comorbidities also affects patients’ treatment responses [12]. However, existing studies only focus on the prognostic value of their coexistence without considering their sequence of development, which may help delineate different disease phenotypes [13,14]. Our team has previously conducted epidemiological studies using a territory-wide diabetes cohort to explore variability in laboratory markers, but not the effects of the comorbidity sequence at baseline for risk prediction [15,16,17]. Therefore, the present study aims to elucidate the effect of the sequence of CHD, AF and HF development on all-cause mortality risk amongst patients with type 2 diabetes mellitus who are on different antidiabetic and cardiovascular medications. 

## 2. Methods

### 2.1. Patient Selection and Data Extraction

This study received ethics approval from the Joint Chinese University of Hong Kong–New Territories East Cluster Clinical Research Ethics Committee. The cohort consists of type 2 diabetes mellitus patients prescribed antidiabetic or cardiovascular medications by the Hong Kong hospital authority between 1 January 2009 to 31 December 2009. The patients were followed-up with from their recruitment date till death or 31 December 2019, whichever was earlier. Demographic, clinical and biochemical data were identified and extracted from the Clinical Data Analysis and Reporting System (CDARS), a Hong Kong-wide electronic healthcare database that compiles data from across all hospitals under the Hong Kong Hospital Authority to form a holistic record of medical data for individual patients. The CDARS has been used by both our team [18,19,20,21] and other teams [22,23,24] in Hong Kong to conduct epidemiological studies. 

Prior comorbidities diagnosed between 1 January 1999 to 31 December 2008 were extracted based on the respective International Classification of Diseases, Ninth Revision (ICD-9) codes in the CDARS documentation (Supplementary Table S1). The sequence of occurrence between AF, CHD and HF was identified based on their difference in the number of days before 1 January 2009. Data on the following classes of antidiabetic agents were extracted: biguanide, sulphonylurea, insulin, thiazolidinedione, meglitinide, dipeptidyl peptidase-4 inhibitors and glucagon-like peptide receptor-1 agonists. The extracted classes of cardiovascular medications include angiotensin-converting enzyme inhibitors (ACEIs)/angiotensin receptor blockers (ARBs), beta-blockers, calcium channel blockers (CCBs) and lipid-lowering agents. Though emerging evidence shows the importance of sodium-glucose cotransporter-2 inhibitors (sGLT2i) in the prevention of cardiovascular mortality, there was insufficient data from following-up patients on sGLT2i within the past 10 years as the medication was only introduced in Hong Kong 3 years ago. As a result, sGLT2i was not included in the analysis. 

### 2.2. Study Outcome and Statistical Analysis

The study outcome was all-cause mortality, which was extracted from the Hong Kong Death Registry. For descriptive statistics, the continuous variables were presented as mean (standard deviation) and the categorical variables were stated as total number (percentage). The incidence rate of all-cause mortality was calculated by dividing the total number of deceased patients by the sum of years in follow-up. Univariable Cox regression was used to evaluate the predictive value of demographic, clinical and biochemical variables. The results were reported in the form of the hazard ratio (HR) (95% confidence interval (CI)). The predictive value of individual antidiabetic and cardiovascular medication use, adjusted for the sequences and combinations of AF, CHD and HF development, were assessed using multivariable Cox regression. Kaplan–Meier curves were constructed for the survival of patients with comorbidities (AF, CHD and HF) and sequential complications of different combinations of comorbidities. Forest plots summarizing the HRs adjusted under different AF/CHD/HF combinations and sequences were stratified based on the medication class examined and the comorbidity involved. Statistical significance is defined as *p* < 0.05. The statistical analysis was conducted using R Studio (version: 1.1.456) and BioRender was used to create our flowchart.

## 3. Results

### 3.1. Baseline Characteristics

**Figure 1:**
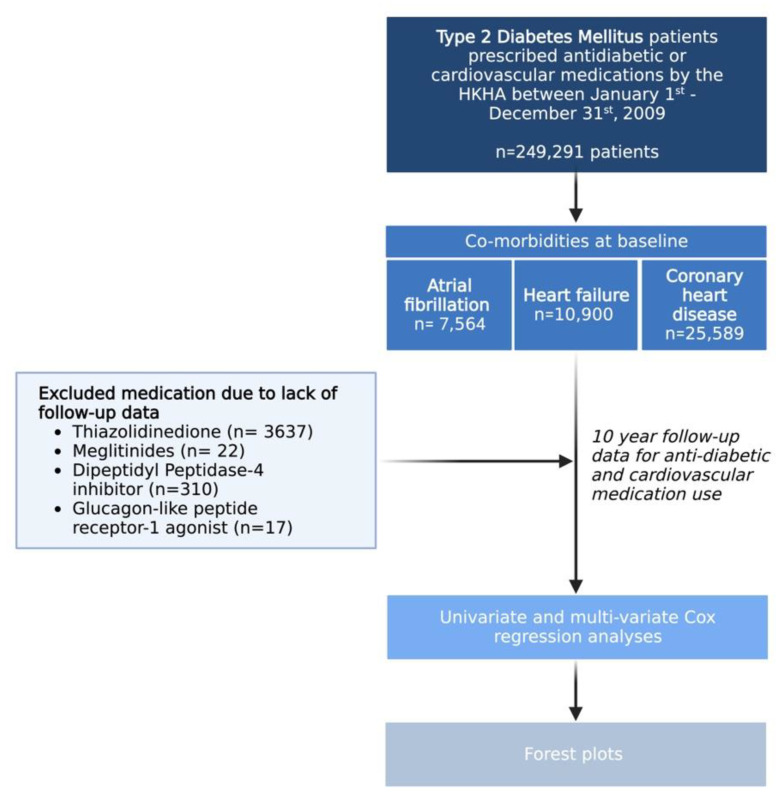
Summary of type 2 diabetes patient cohorts with subgroups categorized by comorbidities and exclusion of certain medication cohorts in the 10-year follow-up.

A total of 249,291 patients (baseline age: 66.0 ± 12.4, 47.4% male) were included. The baseline characteristics of the cohort are summarized in Table 1 and our inclusion algorithm for further analyses is shown in Figure 1. At baseline, 7564, 10,900 and 25,589 patients had a history of AF, HF and CHD, respectively. A total of 170 patients had a history of AF, HF and CHD. Due to the limited availability of more novel antidiabetic agents at the time of follow-up of 10 years, there were few patients prescribed thiazolidinedione (n = 3637), meglitinides (n = 22), dipeptidyl peptidase-4 inhibitors (n = 310) and glucagon-like peptide receptor-1 agonists (n = 17). As a result, these antidiabetic agents were not included in the comorbidity-adjusted Cox regression model. 

### 3.2. Univariable Cox Regression Model 

Over a follow-up of 3524 ± 1218 days, 85,870 patients died, which corresponded to an all-cause mortality rate of 35.7 per 1000 person-years. The findings of the univariable Cox regression are summarized in Table 2. Nonmodifiable risk factors such as older age (HR: 1.09, 95% CI: [1.09–1.09]), *p* < 0.001) and the male gender (HR: 1.13, CI: [1.12–1.15], *p* < 0.001) were significant predictors for all-cause mortality. A history of AF (HR: 3.42, 95% CI: [3.32–3.51], *p* < 0.001), HF (HR: 4.61, 95% CI: [4.51–4.71], *p* < 0.001) and CHD (HR: 2.17, 95% CI: [2.13–2.21], *p* < 0.001) are significant predictors for all-cause mortality. Similarly, the use of antidiabetic and cardiovascular medications are significant univariable Cox predictors as well (*p* < 0.001). Whilst all sequences of AF, HF and CHD were demonstrated to be significant predictors, patients with HF-AF had the highest mortality risk (HR: 5.25, 95% CI: [4.91–5.62], *p* < 0.001), which is much higher than its AF-HF counterpart (HR: 4.58, 95% CI: [4.35–4.82], *p* < 0.001). Similar mortality risks were reported for patients who have a history of AF, HF and CHD. The sequence of CHD-HF-AF reported the highest mortality risk (HR: 6.42, 95% CI: [5.48–7.51], *p* < 0.001). The Kaplan–Meier survival curves also confirmed the significant risk of mortality amongst patients with heart failure, coronary heart disease and atrial fibrillation comorbidities and sequences of complications (Supplementary Figures S1 and S2).

### 3.3. Multivariable Cox Regression of Sequential Complications Adjusted for Age, Sex and Renal Disease

The results from the multivariable Cox regression, demonstrating the comorbidity-adjusted mortality risk of cardiovascular and antidiabetic medications, are summarized in Table 3. Histories of AF (HR: 2.03, 95% CI: [1.98–2.09], *p* < 0.001), HF (HR: 2.42, 95% CI: [2.36–2.47], *p* < 0.001) and CHD (HR: 1.49, 95% CI: [1.46–1.51], *p* < 0.001) were all significant predictors for all-cause mortality. However, medications such as thiazolidinedione (HR: 1.02, 95%CI: [1.46–1.51], *p* = 0.53), meglitinide (HR: 0.92, 95% CI: [0.50–1.72], *p* = 0.80) and glucagon-like receptor peptide-1 agonists (HR: 0.67, 95% CI: [0.17–2.67], *p* = 0.57) were insignificant multivariable Cox predictors. All sequential comorbidities of AF, HF and CHD were significant predictors, with combinations of HF-AF (HR: 2.55, 95% CI: [2.39–2.73], *p* < 0.001) and CHD-AF-HF (HR: 2.80, 95% CI: [2.55–3.08], *p* < 0.001) having the highest risk of mortality. Interestingly, although we found that the use of most antidiabetic and cardiovascular medications are significant multivariable Cox predictors as well (*p* < 0.001), thiazolidinedione (*p* = 0.53), meglitinide (*p* = 0.80) and glucagon-like receptor peptide-1 agonists (*p* = 0.57) did not reach significance after adjusting for age, sex and renal disease.

### 3.4. Mortality Risk after Adjusting for Comorbidities and Cardiovascular and Antidiabetic Medications

The comorbidity-adjusted mortality risks of cardiovascular and antidiabetic medications are summarized in Table 4. Several findings can be noted for patients on antidiabetic medications. 

Although the development of CHD in isolation amongst biguanide users (HR: 0.49, 95% CI: [0.47–0.51], *p* < 0.001) had a higher mortality risk than their counterparts who only developed AF (HR: 0.48, 95% CI: [0.45–0.50], *p* < 0.001) or HF (HR: 0.37, 95% CI: [0.35–0.38], *p* < 0.001), an earlier presentation of CHD in the presence of multiple comorbidities marks for a better prognosis. For insulin users, the development of CHD earlier in the disease course had a lower mortality risk. Amongst insulin users with two of the three comorbidities, CHD patients with preceding AF (HR: 3.06, 95% CI: [2.60–3.61], *p* < 0.001) or HF (HR: 3.84, 95% CI: [3.47–4.24], *p* < 0.001) had a worse prognosis. For sulphonylurea, the development of CHD later in the disease course conferred with a better prognosis. In the presence of all three comorbidities in sulphonylurea users, the earlier presentation of CHD marked for a worse prognosis, contrarily to the results in biguanide users. However, we found that the development of AF in isolation amongst sulphonylurea users had a higher mortality risk when compared with patients who only developed HF or CHD. Amongst sulphonylurea users with two of the three comorbidities, there were two groups that had the worst prognosis: AF patients with preceding CHD (HR: 1.02, 95% CI: [0.90–1.15], *p* < 0.001) and HF patients with preceding AF (HR: 1.02, 95% CI: [0.91–1.14], *p* < 0.001).

Interestingly, the number of comorbidities and sequence of comorbidity development had little effect on the mortality risk amongst ACEI/ARB and CCB users. However, in ACEI/ARB users with two of the three comorbidities, the risk of mortality was higher in patients with HF as one of the comorbidities compared with patients who did not have HF, irrespective of earlier or later presentations. Similarly, CCB users with only a combination of HF and CHD out of the three comorbidities had a higher risk of mortality. The presence of CHD in the sequence of comorbidities increases the mortality risk for patients on lipid-lowering agents and beta-blockers. In users of lipid-lowering agents who developed all three comorbidities, those with preceding AF had a greater risk of mortality (AF-CHD-HF: HR: 3.22, 95% CI: [2.24–4.61], *p* < 0.001; AF-HF-CHD: HR: 3.71, 95% CI: [2.66–5.16], *p* < 0.001). 

## 4. Discussion

To the best of our knowledge, this is the first study that examines the effect of cardiovascular comorbidity sequences on the risk of mortality. There are several notable findings from the present study: (1) the number of comorbidities and sequence of cardiovascular comorbidity development affect the mortality risk to a varying degree between different medications; (2) the increase in the number of cardiovascular comorbidities does not always translate into an increase in mortality risk, depending on the type of cardiovascular comorbidities involved and the sequence of development; and (3) sequences of cardiovascular comorbidities have a different effect on the mortality risk amongst patients on different medications. 

Although it seems intuitive that the increase in the number of comorbidities directly translates into an increase in mortality risk, existing studies have reported otherwise. A study on the association between guideline-recommended drugs and mortality amongst elderlies with multiple comorbidities reported a similar mortality risk amongst patients with AF, CHD, HF, hyperlipidemia and hypertension, in isolation and combination [25]. In a recent study on the effects of multi-comorbidity on the mortality of patients with chronic obstructive pulmonary disease, it was found that whilst multi-comorbidity is an independent marker for mortality, only some comorbidities affect mortality [26]. Long-term comorbidities that are associated with frailty are more likely to increase the risk of mortality [19,27]. For example, the mortality risk of patients with dementia is increased after adjusting for the comorbidity load [28]. Thus, the earlier intervention of these comorbidities may help to delay the progression of frailty and, therefore, reduce the risk of mortality. 

The variable effects of the comorbidity sequence on the mortality risk of patients prescribed different medications are contributed to by a multitude of factors. For example, patients’ responses to medications vary based on the presence of different comorbidities. Unlike HF patients who are in sinus rhythm, the efficacy of beta-blockers in HF patients with AF remained controversial in terms of the benefits on survival [29]. It has been reported that amongst women on beta-blockers with CHD, there is an increased risk of new-onset HF, which is associated with increased 30-day mortality [30]. Moreover, the difference in the indication of medication prescription between diseases may have contributed to the difference in mortality risk. A recent meta-analysis on more than 100,000 AF patients reported that statin therapy can result in a 10% absolute reduction in all-cause mortality risk [31]. However, unlike CHD and HF, AF patients were not routinely started on lipid-lowering therapies under the current guidelines. The delay in treatment initiation may have resulted in a greater risk of mortality in AF patients who subsequently developed other cardiovascular complications. 

The sequence of comorbidity development is both a marker of disease severity and the window of opportunity for intervention to improve patient outcomes. In the present study, the development of CHD earlier in the disease course marks a lower mortality risk amongst insulin users, but a higher risk amongst sulphonylurea users. Since insulin is prescribed to patients with more advanced diabetes mellitus, an earlier diagnosis of CHD allows for early intervention and the potential early diagnosis of other cardiovascular complications, thus improving their overall survival. Similar findings were demonstrated in the higher mortality risk amongst first-presentation ST-elevation myocardial infarction patients free of modifiable cardiovascular risk factors than their counterparts with pre-existing risk factors, where the risks were attenuated after adjusting for the application of guideline-directed therapy [32]. Current studies that demonstrate higher cardiovascular adverse events and mortality risk amongst insulin users randomized patients with diabetes mellitus into insulin provision and insulin sensitization groups, which did not reflect the more advanced disease state amongst typical insulin users in real life [33,34]. However, given sulphonylurea is a second-line agent, the earlier development of CHD marks for early macrovascular involvement, therefore reflecting a poorer disease prognosis. In addition, the cardioprotective effect of sulphonylurea is relatively weak compared to other antidiabetic agents, and hence, further increases the mortality risk [35,36]. Indeed, many observational studies have reported higher risks of adverse cardiovascular events in sulphonylurea users [21,37,38]. 

Interestingly, many international guidelines have included the use of biguanides and glucose-lowering drugs in the treatment of cardiovascular disease [1,39,40]. The recent literature suggests that the use of empagliflozin, liraglutide and semaglutide correlated with a reduction in mortality and cardiovascular events for patients with pre-existing cardiovascular disease and diabetes [41]. To corroborate, biguanide demonstrated superior preventive effects against cardiovascular risk compared to sulfonylurea in a Japanese diabetic cohort [42]. The beneficial effects of biguanides in the reducing risk of ischemic heart disease, stroke and heart failure are further supported in a Danish case–control study [43]. However, further investigations of the additive effects of glucose-lowering agents are warranted to validate these findings. 

Given the shared risk factors for and pathophysiological mechanisms underlying CHD, AF and HF, it is common for the three conditions to coexist and create a vicious cycle of deterioration amongst patients [44]. Studies have reported that there is a greater increase in mortality risk amongst AF patients with HF of an ischemic origin. Since AF has been reported to increase oxidative stress and endothelial dysfunction [45,46], it exacerbates the myocardial ischemia under the already reduced coronary perfusion in CHD, resulting in more severe HF and earlier mortality [47]. By contrast, atrial ischemia promotes the generation of arrhythmogenic substrates in the atrium, resulting in the development of AF [48]. AF itself is a known risk factor for acute myocardial infarction due to increased myocardial oxygen consumption and adverse cardiac remodeling [49,50,51]. A more holistic understanding of the sequence of complications can guide clinical recommendations in cardiovascular prevention and create more personalized medical regimens for type 2 diabetes. Given that the sequence of complications can be a marker for disease severity, pharmacological measures can account for the variability in mortality risk amongst patients. Our study further supports the need to understand the extent to which the sequence of the cardiovascular complication development affects patient prognosis. 

### Limitations

Several limitations should be noted in the present study. Firstly, the observational nature of the present study makes it susceptible to documentation errors. Secondly, potential treatment noncompliance affects the accuracy in the use of medication prescription as a surrogate marker for medication exposure. Thirdly, due to limitations of the electronic healthcare database, there is a lack of electrocardiographic and echocardiographic data that can be used for patient prognostication. Additionally, this database included mostly patients who are ethnically Han Chinese. Finally, data on lifestyle cardiovascular risk factors such as obesity, smoking and alcoholism were unavailable.

## 5. Conclusions

To conclude, the sequence of comorbidity development affects the risk of all-cause mortality to varying degrees in users of different cardiovascular and antidiabetic agents. Further studies are needed to elucidate the underlying mechanisms and modifications to current management guidelines to improve patient prognosis.

## Figures and Tables

**Table 1:** Baseline characteristics of the present cohort.

Parameter	Total Number (%)/ Mean (Standard Deviation)
Demographic	
Age	66.04 (12.44)
Male	118,262 (47.44)
All-cause mortality	85,870 (34.45)
Medications	
Biguanide	185,069 (74.24)
Sulphonylurea	172,779 (69.31)
Insulin	29,401 (11.79)
Thiazolidinedione	3637 (1.46)
Meglitinide	22 (0.01)
Dipeptidyl peptidase-4 inhibitors	310 (0.12)
Glucagon-like receptor peptide-1 agonists	17 (0.01)
Angiotensin-converting enzyme inhibitors/angiotensin receptor blockers	120,604 (48.38)
Beta-blockers	90,908 (36.47)
Calcium channel blockers	107,879 (43.27)
Lipid-lowering agents	60,152 (24.13)
Comorbidities	
Coronary heart disease (CHD)	25,589 (10.26)
Atrial fibrillation (AF)	7564 (3.03)
Heart failure (HF)	10,900 (4.37)
CHD-HF	3586 (1.44)
CHD-AF	1677 (0.67)
HF-CHD	1919 (0.77)
HF-AF	967 (0.39)
AF-CHD	771 (0.31)
AF-HF	1809 (0.73)
CHD-HF-AF	170 (0.07)
CHD-AF-HF	480 (0.19)
HF-CHD-AF	192 (0.08)
AF-CHD-HF	126 (0.05)
HF-AF-CHD	134 (0.05)
AF-HF-CHD	164 (0.07)
Renal diabetic complications	3323 (1.33)
Peripheral vascular disease	341 (0.14)
Neurological diabetic complications	1144 (0.46)
Ophthalmological diabetic complications	3490 (1.40)
Ischemic stroke and transient ischemic attack	8733 (3.50)
Intracranial hemorrhage	3134 (1.26)
Osteoporosis	134 (0.05)
Dementia	2725 (1.09)
Hypertension	62,571 (25.10)
Chronic obstructive pulmonary disease	793 (0.32)
Cancer	11,442 (4.59)

**Table 2:** Univariable Cox regression to identify significant risk factors for all-cause mortality.

Parameter	Hazard Ratio (95% Confidence Interval)	*p*-Value
Demographic		
Age	1.09 (1.09−1.09)	<0.001
Male	1.13 (1.12−1.15)	<0.001
Medications		
Biguanide	0.58 (0.57−0.58)	<0.001
Sulphonylurea	1.28 (1.26−1.30)	<0.001
Insulin	1.94 (1.91−1.97)	<0.001
Thiazolidinedione	0.79 (0.74−0.83)	<0.001
Meglitinide	1.48 (0.80−2.75)	<0.001
Dipeptidyl peptidase-4 inhibitors	0.48 (0.37−0.62)	<0.001
Glucagon-like receptor peptide-1 agonists	0.30 (0.07−1.19)	<0.001
Angiotensin converting enzyme inhibitors/angiotensin receptor blockers	1.43 (1.41−1.45)	<0.001
Beta-blockers	1.32 (1.31−1.34)	<0.001
Calcium channel blockers	1.82 (1.80−1.85)	<0.001
Lipid-lowering agents	1.30 (1.28−1.32)	<0.001
Comorbidities		
Coronary heart disease (CHD)	2.17 (2.13−2.21)	<0.001
Atrial fibrillation (AF)	3.42 (3.32−3.51)	<0.001
Heart failure (HF)	4.61 (4.51−4.71)	<0.001
CHD-HF	4.66 (4.49−4.83)	<0.001
CHD-AF	4.00 (3.79−4.22)	<0.001
HF-CHD	4.61 (4.39−4.84)	<0.001
HF-AF	5.25 (4.91−5.62)	<0.001
AF-CHD	4.05 (3.74−4.39)	<0.001
AF-HF	4.58 (4.35−4.82)	<0.001
CHD-HF-AF	6.42 (5.48−7.51)	<0.001
CHD-AF-HF	5.34 (4.86−5.87)	<0.001
HF-CHD-AF	5.87 (5.06−6.82)	<0.001
AF-CHD-HF	5.24 (4.35−6.32)	<0.001
HF-AF-CHD	5.55 (4.65−6.63)	<0.001
AF-HF-CHD	5.51 (4.69−6.48)	<0.001
Renal diabetic complications	3.54 (3.40−3.68)	<0.001
Peripheral vascular disease	4.25 (3.79−4.78)	<0.001
Neurological diabetic complications	2.95 (2.76−3.16)	<0.001
Ophthalmological diabetic complications	2.62 (2.51−2.73)	<0.001
Ischemic stroke and transient ischemic attack	2.74 (2.67−2.82)	<0.001
Intracranial hemorrhage	2.60 (2.49−2.71)	<0.001
Osteoporosis	2.77 (2.25−3.40)	<0.001
Dementia	5.66 (5.43−5.89)	<0.001
Hypertension	2.47 (2.43−2.50)	<0.001
Chronic obstructive pulmonary disease	4.43 (4.10−4.79)	<0.001
Cancer	2.39 (2.33−2.45)	<0.001

**Table 3:** Multivariable Cox regression to identify significant risk factors for all-cause mortality adjusting for age, sex and renal disease.

Parameter	Hazard Ratio (95% Confidence Interval)	*p*-Value
Medications		
Biguanide	0.75 (0.74−0.77)	<0.001
Sulphonylurea	1.18 (1.16−1.20)	<0.001
Insulin	1.98 (1.95−2.02)	<0.001
Thiazolidinedione	1.02 (0.96−1.08)	0.53
Meglitinide	0.92 (0.50−1.72)	0.80
Dipeptidyl peptidase-4 inhibitors	0.63 (0.49−0.82)	<0.001
Glucagon-like receptor peptide-1 agonists	0.67 (0.17−2.67)	0.57
Angiotensin converting enzyme inhibitors/angiotensin receptor blockers	1.25 (1.23−1.26)	<0.001
Beta-blockers	1.17 (1.15−1.19)	<0.001
Calcium channel blockers	1.26 (1.24−1.27)	<0.001
Lipid-lowering agents	1.22 (1.20−1.24)	<0.001
Comorbidities		
Coronary heart disease (CHD)	1.49 (1.46−1.51)	<0.001
Atrial fibrillation (AF)	2.03 (1.98−2.09)	<0.001
Heart failure (HF)	2.42 (2.36−2.47)	<0.001
CHD-HF	2.38 (2.29−2.47)	<0.001
CHD-AF	2.18 (2.07−2.30)	<0.001
HF-CHD	2.31 (2.20−2.43)	<0.001
HF-AF	2.55 (2.39−2.73)	<0.001
AF-CHD	2.03 (1.87−2.19)	<0.001
AF-HF	2.44 (2.32−2.57)	<0.001
CHD-HF-AF	2.65 (2.27−3.11)	<0.001
CHD-AF-HF	2.80 (2.55−3.08)	<0.001
HF-CHD-AF	2.77 (2.39−3.22)	<0.001
AF-CHD-HF	2.10 (1.74−2.53)	<0.001
HF-AF-CHD	2.03 (1.70−2.42)	<0.001
AF-HF-CHD	2.81 (2.39−3.31)	<0.001
Peripheral vascular disease	1.80 (1.59−2.02)	<0.001
Neurological diabetic complications	1.42 (1.32−1.53)	<0.001
Ophthalmological diabetic complications	1.93 (1.83−2.02)	<0.001
Ischemic stroke and transient ischemic attack	1.72 (1.67−1.76)	<0.001
Intracranial hemorrhage	1.35 (1.10−1.66)	<0.001
Osteoporosis	2.41 (2.32−2.51)	<0.001
Dementia	1.67 (1.64−1.69)	<0.001
Hypertension	2.06 (1.91−2.23)	<0.001
Chronic obstructive pulmonary disease	1.73 (1.69−1.77)	<0.001
Cancer	1.35 (1.10−1.66)	<0.001

**Table 4:** Comorbidity-adjusted mortality risk of antidiabetic and cardiovascular medications.

Comorbidities	Biguanide	Sulphonylurea	Insulin	ACEIs/ARBs	Beta-Blockers	CCBs	Lipid-Lowering Agents
CHD	0.49 (0.47−0.51)	1.01 (0.98−1.05)	2.06 (1.98−2.13)	1.63 (1.57−1.69)	1.93 (1.86−2.00)	1.06 (1.02−1.09)	2.50 (2.42−2.59)
AF	0.48 (0.45−0.50)	1.03 (0.97−1.09)	1.45 (1.36−1.54)	1.57 (1.49−1.66)	1.36 (1.29−1.43)	1.00 (0.95−1.05)	1.31 (1.24−1.38)
HF	0.37 (0.35−0.38)	0.89 (0.85−0.93)	2.31 (2.21−2.41)	2.19 (2.09−2.30)	1.49 (1.43−1.56)	0.95 (0.91−0.99)	1.93 (1.85−2.01)
CHD-HF	0.30 (0.27−0.32)	0.95 (0.88−1.03)	2.47 (2.29−2.67)	2.70 (2.48−2.94)	2.21 (2.05−2.38)	0.78 (0.73−0.84)	3.89 (3.61−4.19)
CHD-AF	0.38 (0.34−0.42)	1.02 (0.90−1.15)	1.46 (1.28−1.66)	2.30 (2.04−2.60)	1.81 (1.62−2.01)	0.86 (0.78−0.96)	2.62 (2.35−2.91)
HF-CHD	0.39 (0.35−0.43)	0.75 (0.67−0.83)	3.84 (3.47−4.24)	2.65 (2.36−2.99)	2.24 (2.03−2.48)	0.84 (0.76−0.92)	3.81 (3.45−4.22)
HF-AF	0.44 (0.38−0.50)	0.90 (0.78−1.05)	2.45 (2.12−2.83)	2.47 (2.11−2.89)	1.44 (1.26−1.65)	0.88 (0.77−1.01)	1.46 (1.27−1.68)
AF-CHD	0.48 (0.41−0.56)	0.79 (0.67−0.94)	3.06 (2.60−3.61)	2.19 (1.83−2.63)	1.94 (1.65−2.28)	1.05 (0.90−1.24)	2.67 (2.28−3.13)
AF-HF	0.35 (0.32−0.39)	1.02 (0.91−1.14)	1.51 (1.34−1.70)	2.48 (2.20−2.79)	1.33 (1.21−1.47)	0.85 (0.77−0.94)	1.57 (1.41−1.74)
CHD-HF-AF	0.30 (0.22−0.41)	1.09 (0.76−1.56)	2.39 (1.70−3.36)	3.12 (2.11−4.63)	1.91 (1.39−2.63)	0.76 (0.55−1.04)	2.65 (1.94−3.64)
CHD-AF-HF	0.28 (0.23−0.34)	1.16 (0.93−1.44)	1.24 (0.98−1.58)	2.89 (2.29−3.64)	1.61 (1.33−1.94)	0.74 (0.61−0.89)	2.61 (2.16−3.16)
HF-CHD-AF	0.37 (0.27−0.50)	0.72 (0.52−0.98)	3.03 (2.23−4.13)	2.19 (1.56−3.08)	1.86 (1.37−2.52)	0.86 (0.64−1.16)	2.24 (1.66−3.02)
AF-CHD-HF	0.36 (0.25−0.52)	0.94 (0.64−1.40)	4.63 (3.24−6.63)	2.40 (1.58−3.64)	2.32 (1.60−3.36)	0.85 (0.60−1.22)	3.22 (2.24−4.61)
HF-AF-CHD	0.46 (0.31−0.66)	0.74 (0.50−1.10)	3.13 (2.13−4.60)	4.29 (2.56−7.19)	1.28 (0.88−1.86)	1.01 (0.69−1.46)	2.49 (1.71−3.62)
AF-HF-CHD	0.42 (0.30−0.58)	0.80 (0.56−1.13)	3.18 (2.27−4.44)	2.68 (1.82−3.95)	1.78 (1.28−2.46)	1.10 (0.79−1.53)	3.71 (2.66−5.16)

ACEIs/ARBs: angiotensin-converting enzyme inhibitors/angiotensin receptor blockers; CCBs: calcium channel blockers.

## Data Availability

Anonymized data available upon request to the corresponding authors.

## Supplementary Materials

The following supporting information can be downloaded at: https://www.mdpi.com/article/10.3390/life12121956/s1, Figure S1: Kaplan-Meier Survival Analysis on the effects of atrial fibrillation, coronary heart disease, and heart failure on mortality; Figure S2: Kaplan-Meier survival plot for survival in days in sub-groups divided by sequential complications of heart failure, coronary artery disease, and atrial fibrillation; Table S1: ICD-9 Codes of the comorbidities.
