# Supplementary material for: The Impact of Cardiac Comorbidity Sequence at Baseline and Mortality Risk in Type 2 Diabetes Mellitus: A Retrospective Population-Based Cohort Study
Source: Life (Basel). 2022 Nov 22;12(12):1956. doi: 10.3390/life12121956 (PMC9781363; doi:10.3390/life12121956)
Supplement: Supplementary file 1 [file life-12-01956-s001.zip › life-1988374-supplementary.pdf]

Supplementary Table S1. ICD-9 Codes of the comorbidities.

Comorbidities	ICD Code
Ophthalmological Diabetic Complications	250.5
Renal Diabetic Complications	250.4
Neurological Diabetic Complications	250.6
Peripheral Vascular Disease	250.7
Ischemic Stroke and Transient Ischemic Attack	433.01, 433.11, 433.21, 433.31, 433.81, 433.91, 434.01, 434.91, 436, 435
Intracranial Hemorrhage	430.00, 431.00, 432.00, 432.10, 432.90, 852.00-852.09, 852.20-852.29, 852.40-852.49, 853.00
Atrial Fibrillation	427.31, 429.4
Heart Failure	428.00-428.99
Coronary Heart Disease	410.00-411.99, 413.00-414.99
Osteoporosis	733.00
Dementia	290.00-290.99, 331.00, 294.10-294.29, 331.19
Chronic Obstructive Pulmonary Disease	490.00-496.99
Hypertension	491.00-492.99
Cancer	140.00-209.99

Supplementary Figure S1. Kaplan-Meier Survival Analysis on the effects of atrial fibrillation, coronary heart disease, and heart failure on mortality

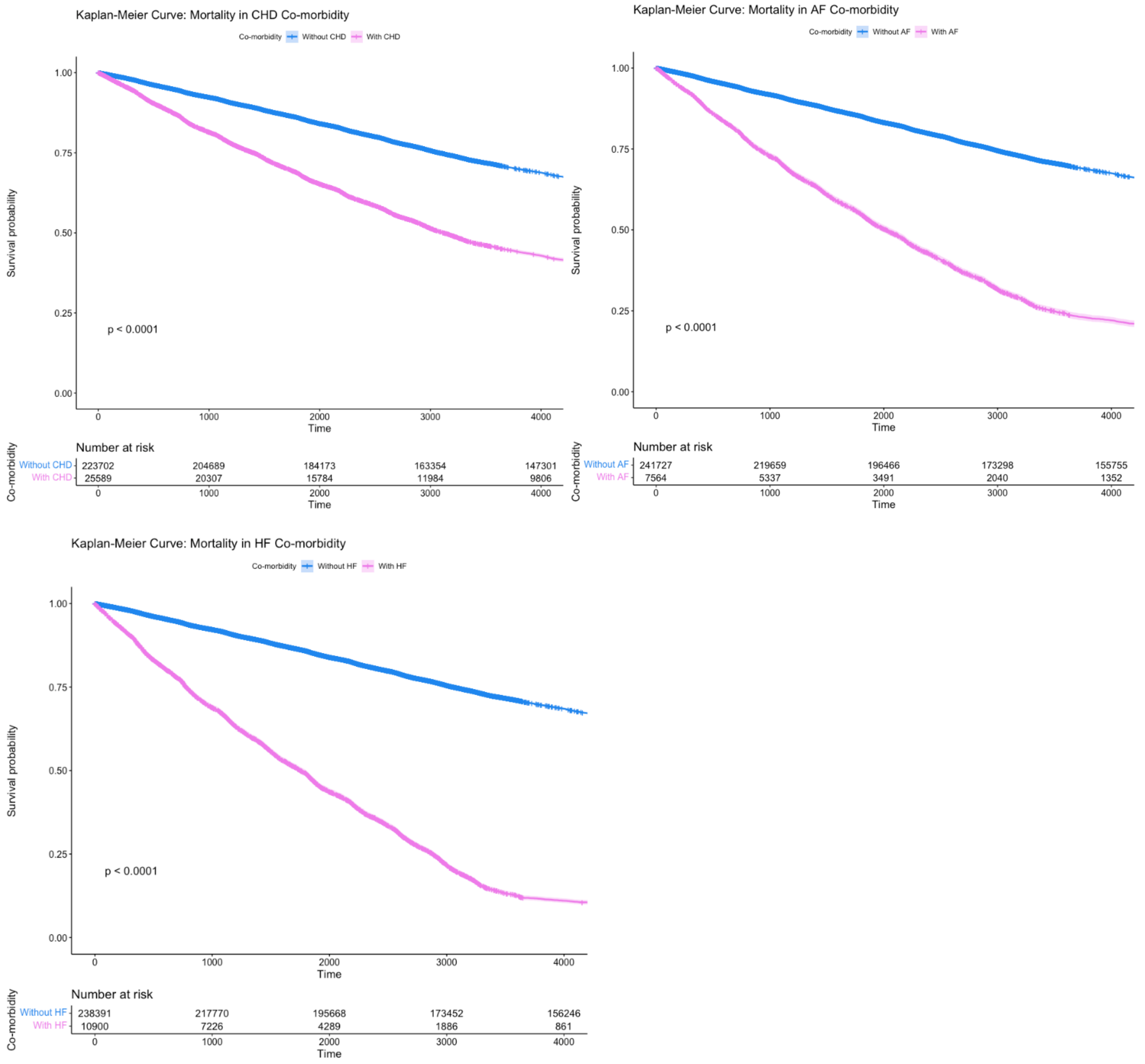

Supplementary Figure S2. Kaplan-Meier survival plot for survival in days in sub-groups divided by sequential complications of heart failure, coronary artery disease, and atrial fibrillation.

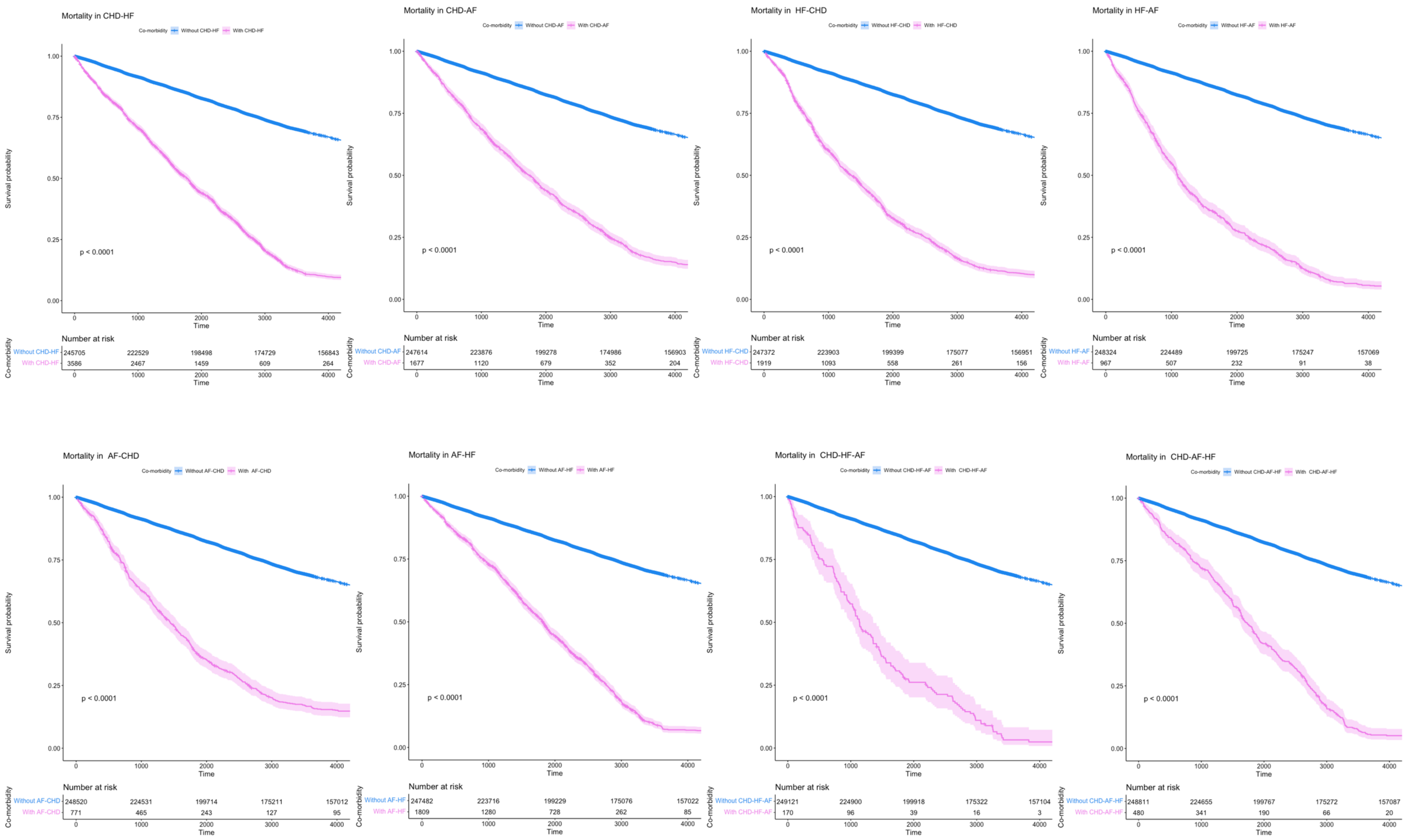

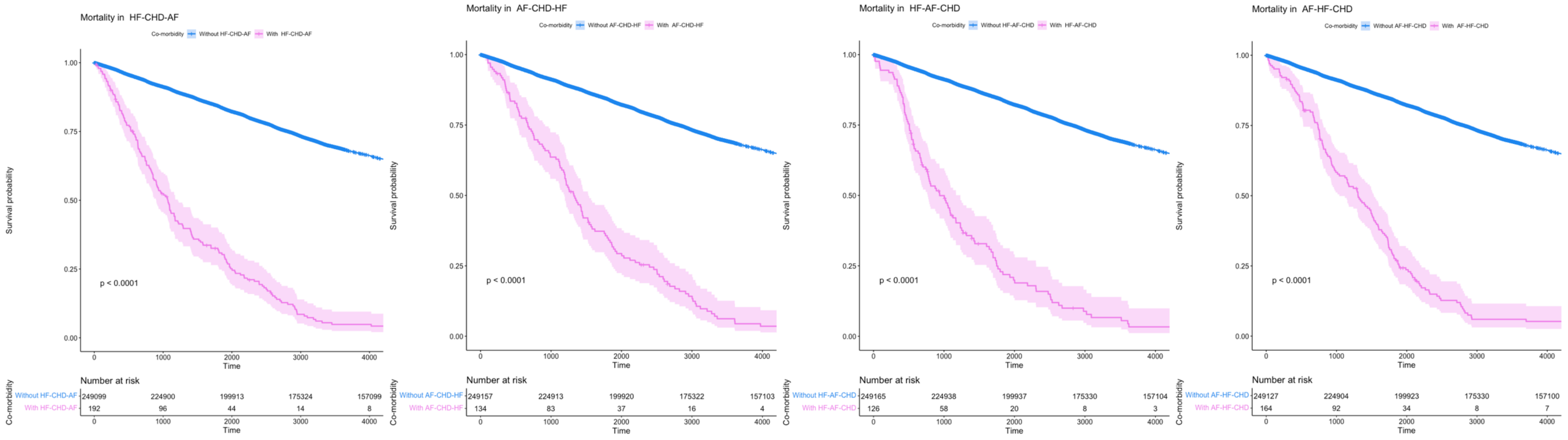
Mortality in HF-CHD-AF
Co-morbidity Without HF-CHD-AF With HF-CHD-AF
Survival probability
p < 0.0001
Time
Number at risk
Without HF-CHD-AF 249099 224900 199913 175324 157099
With HF-CHD-AF 192 96 44 14 8
Mortality in AF-CHD-HF
Co-morbidity Without AF-CHD-HF With AF-CHD-HF
p < 0.0001
Number at risk
Without AF-CHD-HF 249157 224913 199920 175322 157103
With AF-CHD-HF 134 83 37 16 4
Mortality in HF-AF-CHD
Co-morbidity Without HF-AF-CHD With HF-AF-CHD
p < 0.0001
Number at risk
Without HF-AF-CHD 249165 224938 199937 175330 157104
With HF-AF-CHD 126 58 20 8 3
Mortality in AF-HF-CHD
Co-morbidity Without AF-HF-CHD With AF-HF-CHD
p < 0.0001
Number at risk
Without AF-HF-CHD 249127 224904 199923 175330 157100
With AF-HF-CHD 164 92 34 8 7
Co-morbidity
